# A new species of the genus *Policordia* (Bivalvia, Verticordioidea, Lyonsiellidae) from off the coast of southern California

**DOI:** 10.3897/zookeys.622.9411

**Published:** 2016-10-06

**Authors:** Lyudmila A. Safonova, Kelvin L. Barwick

**Affiliations:** 1 Department of Invertebrate Zoology, Biological Faculty, Moscow State University, Moscow 119992, Russia; 2 Orange County Sanitation District, 10844 Ellis Avenue, Fountain Valley, California 92708, USA

**Keywords:** Policordia hispida, Heterodonta, Anomalodesmata, Eastern Pacific, carnivorous bivalves, bathyal

## Abstract

A new species, *Policordia hispida*, is described and compared with three similar species: *Policordia densicostata* (Locard, 1898); *Policordia pilula* (Pelseneer, 1911) and a yet un-described species, *Policordia* sp. (= *Policordia pilula* sensu Ivanova, 1977 not Pelseneer, 1911). This is a first record for the genus in the Californian province.

## Introduction

Like other lyonsiellids, the genus *Policordia* Dall, Bartsch & Rehder, 1938 (Bivalvia, Lyonsiellidae) comprises specialized carnivorous bivalves widely distributed in the world’s oceans. Representatives of the genus live in a large range of depths, 138–9380 m (Allen and Turner 1974; Knudsen 1970) but most commonly occur in deep-sea ocean basins and trenches. *Policordia* is characterized by having a small thin fragile shell with an external sculpture consisting of very fine commarginal growth lines and radial threads. It lacks hinge teeth (Coan et al. 2000). It differs from other genera of the family Lyonsiellidae in that *Policordia* lacks any granules or spinules on the shell surface (Allen and Turner 1974; Poutiers and Bernard 1995). Anatomical modifications of the digestive and respiratory systems of *Policordia* are the result of adaptations for a carnivorous way of life. The digestive tract of *Policordia* includes a muscular stomach with an inner layer of scleroprotein used to crush prey. The stomach is connected to extensive digestive diverticula by one or two ducts. Members of *Policordia* have a large inhalant siphon surrounded by sensitive tentacles. Ctenidia are present but reduced, varying in their filament numbers and the degree of their muscularization among species (Allen and Turner 1974; Ivanova 1977).

Presently 25 species are assigned to the genus *Policordia* primarily on the basis of conchological features (Bouchet and Gofas 2013). However, data on anatomical characters show that diversity in the genus is much higher and *Policordia* may prove to be a paraphyletic group requiring thorough revision (Safonova 2007).

Recently specimens conchologically similar to the type species of the genus (*Policordia diomedea* Dall, Bartsch & Rehder, 1938) were collected from two separate sites off the coast of southern California. These are the first records of the genus from the Californian Marine Province (Coan et al. 2000). Here we describe it as a new species.

## Methods

The new species is represented by preserved, live taken, specimens that were collected using a 0.1 m^2^ chain-fired Van Veen Grab. Sediment samples were screened with a 1 mm sieve, fixed in a 10% solution of buffered formaldehyde and then transferred to 70% ethanol. Morphological measurements were made with calipers and an ocular micrometer (±0.1 mm). The length (L), height (H) and width (W) of the valves were recorded. Gross anatomy was observed using a dissecting microscope.

**Additional material used.** RV “Vityaz”, Cruise 45, station 6103, 59.1167°N, 142.1°W; 1500 m, 1 specimen; 11 May 1969 (*Policordia* sp. = *Policordia pilula* of Ivanova, 1977 not Pelseneer, 1911, deposited in IORAS).

**Institutional abbreviations**:

LACM
Museum of Natural History of Los Angeles County 


SBMNH
Santa Barbara Museum of Natural History



CSD-EMTS
 City of San Diego Environmental Monitoring Technical Services Laboratory 


OCSD
 Orange County Sanitation District 


IBS RAS
 A.V. Zhirmunsky Institute of Marine Biology, Vladivostok, Russia 


IO RAS
P. P. Shirshov Institute of Oceanology, Moscow, Russia 


## Systematic account

### Superfamily Verticordioidea Stoliczka, 1870 Family Lyonsiellidae Dall, 1895

#### Policordia

Taxon classificationAnimaliaAnomalodesmataLyonsiellidae

Dall, Bartsch & Rehder, 1938

##### Type species.

By original designation, *Policordia diomedea* Dall, Bartsch & Rehder, 1938.

##### Recent.

Atlantic, Indian and Pacific Oceans.

##### Gender.

Feminine.

#### Policordia hispida sp. n.

Taxon classificationAnimaliaAnomalodesmataLyonsiellidae

http://zoobank.org/95DD6BCE-3EB5-49B2-8C27-FCB685CDF25D

##### Type locality.

USA, California, Orange County; 33.3688°N; 117.6899°W; 411 m (OCSD B13-9137; 30 July 2013).

##### Type material.

***Holotype*:** LACM 3322, valves separated, with soft parts in 70% ethanol, length 4.8 mm, height 4.3 mm, width 1.8 mm. ***Paratype***: SBMNH 462739, USA, California, San Diego County; 32.7993°N; 117.4055°W; 449 m (CSD-EMTS 8338, 23 July 2014); valves separated, with soft parts in 70% ethanol, length 3.8 mm, height 3.4 mm, width 1.4 mm.

##### Diagnosis.

Shell subquadrate, longer than high with broadly rounded posterodorsal margin; 30–32 radial periostracal lamellae present; umbones prominent. Each mantle margin with approximately 30 mantle glands lacking long cylindrical portion. Inhalant siphon with 18–19 papillated tentacles and 2 pairs of smooth tentacles. Exhalant siphon with 5 conical tentacles. Byssal thread present.

##### Description.

**Figure 1.**
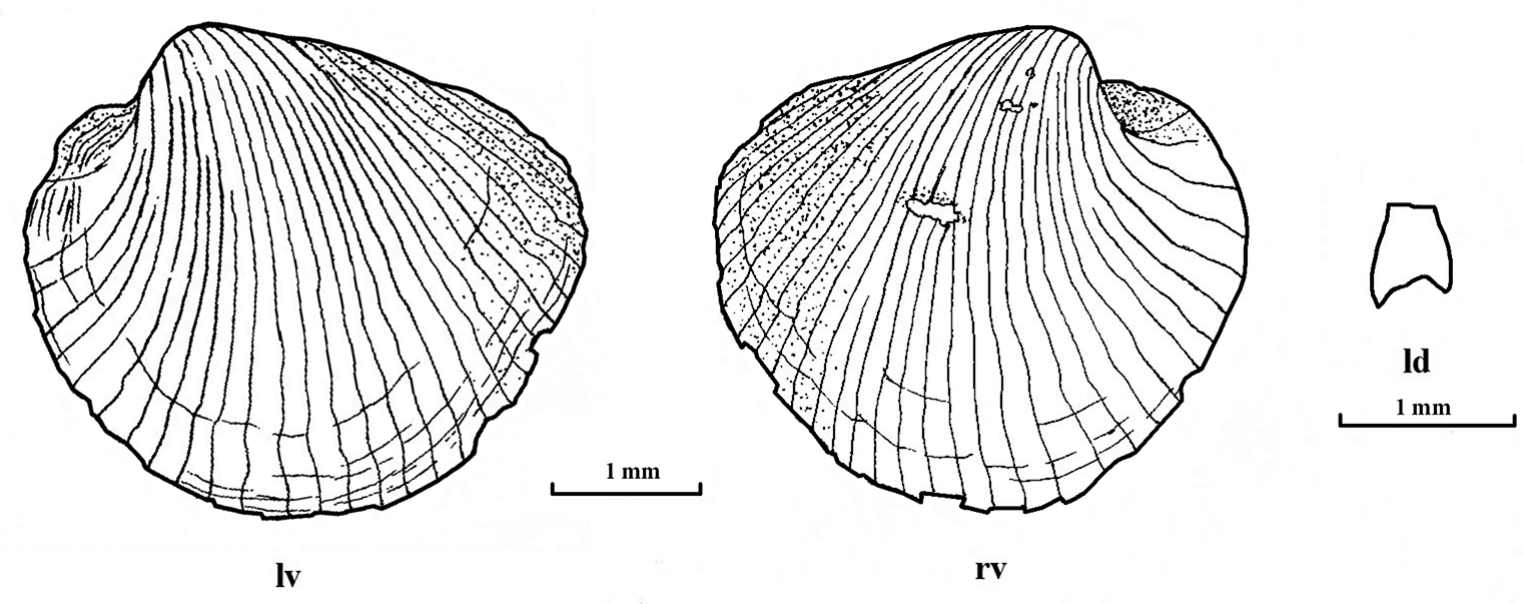
*Policordia hispida* sp. n. Shell of holotype, external view and lithodesma; **lv** left valve **rv** right valve **ld** lithodesma.

***Shell*.** The translucent fragile shell is subquadrate (Figs 1, 3) with the length greater than the height (Table 1); inequivalve, with the right valve overlapping the left. The beaks are prosogyrate, delimited from the posterior angle by compressions. The posterodorsal margin is straight and is directed obliquely downwards from umbo; the posteroventral margin is slightly rounded and forms extended and slightly compressed angle with the posterodorsal margin. The anteroventral margin is rounded on the left valve and nearly straight on the right valve. Shell with irregular fine commarginal growth checks, covered with a colorless periostracum, which in turn forms a series of regularly spaced, radial lamellae (30–32) extending from the umbo, of the total, 13–14 are secondary (incomplete). Adhering to some radial lamellae are bunches of fine fibers giving the shell a slightly hirsute appearance externally. Shell nearly completely covered with fragile thick-silt coating (not shown, removed prior to examination). Hinge margin thin, edentate, with relatively large lithodesma inserted posterior to umbo along posterodorsal margin. Lithodesma has small posterior sinus, about ¼ of length, right posterior branch less than the left (Fig. 1).

**Table 1.** Measurement of right valve (mm).

Measurement	L	H	W	H/L	W/L
Holotype (LACM 3322)	4.8	4.3	1.8	0.9	0.4
Paratype (SBMNH 462739)	3.8	3.4	1.4	0.9	0.4

**Figure 2.**
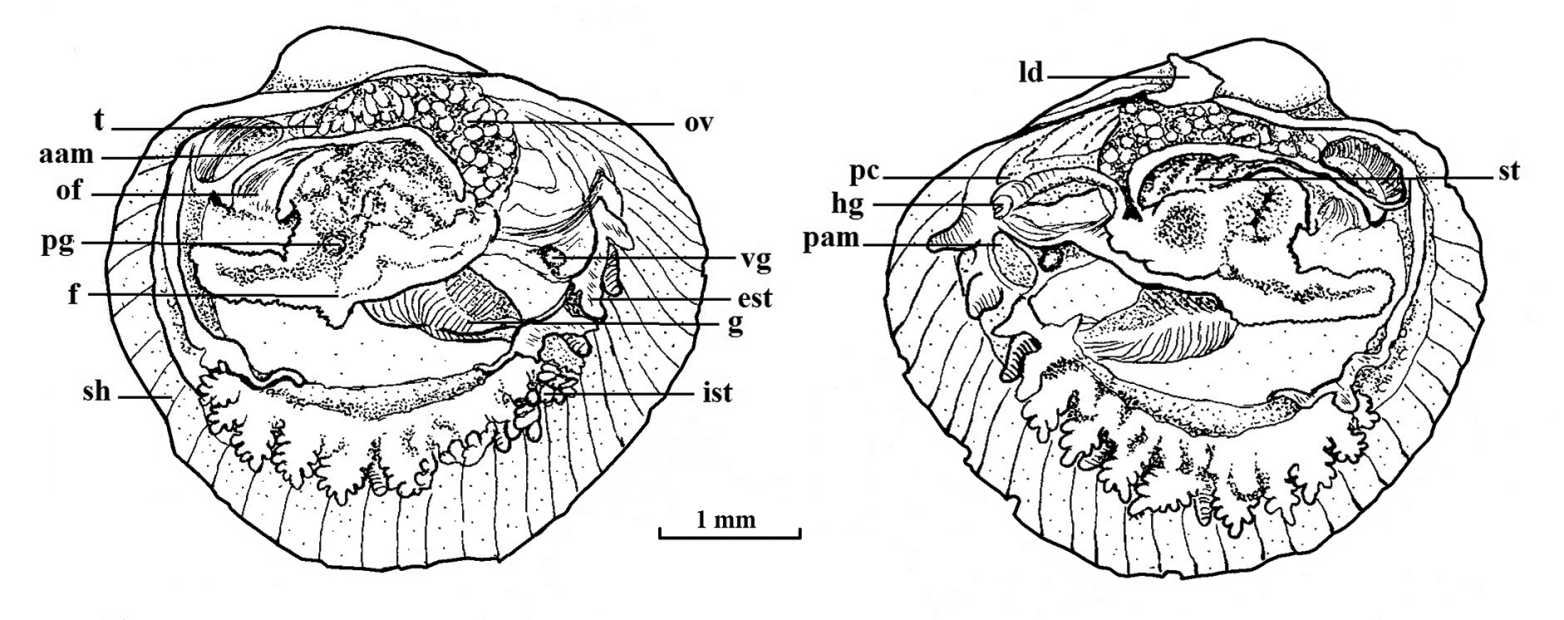
*Policordia hispida* sp. n. Body structure, medial section through the body (views from left and right respectively); **aam** anterior adductor muscle **est** exhalant siphon tentacles **f** foot **g** gills **hg** hindgut **ist** inhalant siphon tentacles **ld** lithodesma **of** oral funnel **ov** ovaries **pam** posterior adductor muscle **pc** pericardium **pg** pedal ganglion **sh** shell **st** stomach **t** testis **vg** visceral ganglion.

**Figure 3.**
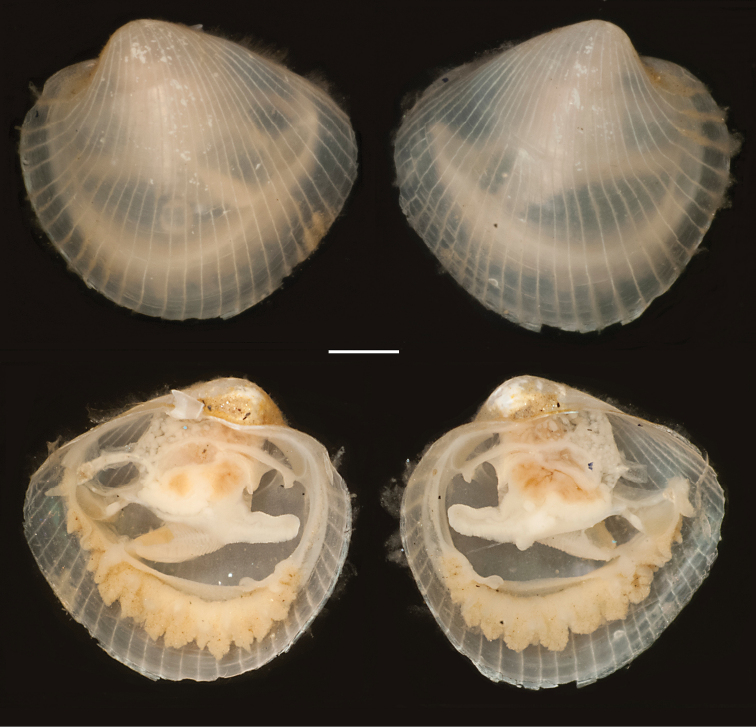
*Policordia hispida* sp. n. Holotype, wet specimens in 70% ethanol; left and right valve respectively; scale bar = 1 mm.

**Figure 4.**
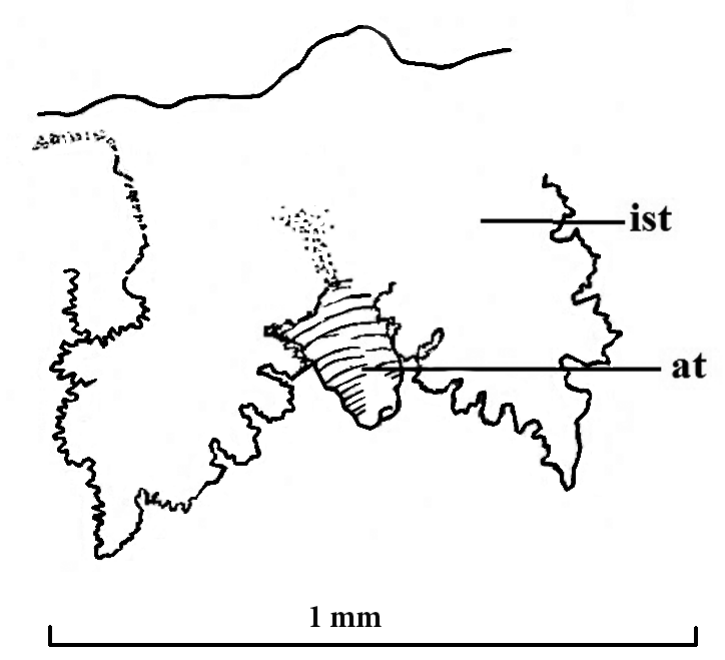
*Policordia hispida* sp. n. The fifth and sixth papillated tentacles of the inhalant siphon with a simple conical tentacle between them; **ist** inhalant siphon tentacles **at** conical tentacle.

***Anatomy*.** Mantle of the holotype has about 30 flask-shaped mantle glands located along the mantle edge. Mantle glands consist of short ducts formed by a few cells and oval structure above them, similar to glands of *Policordia atlantica* (Allen and Turner 1974). There is one row of papillated tentacles surrounding the inhalant siphon, 10 tentacles on the right side and 9 on the left (Figs 2, 3). Each tentacle carries 6–8 short papillated extensions (Figs 2, 4). Additionally, slightly outside the row of papillated tentacles, there are two, left and right, pairs of simple conical tentacles (Fig. 4). The first pair (counting from anterior to posterior) is between the fifth and sixth papillated tentacles; the second pair is between the seventh and eighth papillated tentacles. The exhalant siphon is surrounded by a total of five conical tentacles, one located dorsally and two pairs laterally (Figs 2, 3).

The foot has a heel; byssus present (Figs 2, 3).

**Figure 5.**
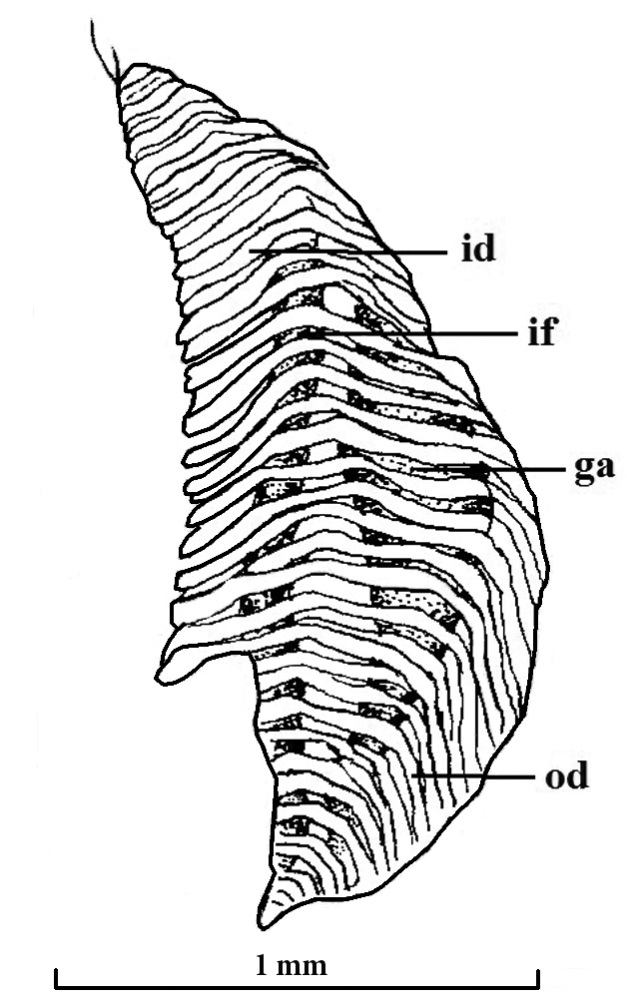
*Policordia hispida* sp. n. Left gill, ventral view; **ga** gill axis **id** inner demibranch **if** interfilamentar partition **od** outer demibranch.

Gills comparably wide, elongated, longer than base of foot. They consist of inner and outer demibranchs (Fig. 5); attached by outer demibranch margin laterally to mantle wall and posteriorly to junction between siphons. Outer demibranch without inter-filamentary junctions, inner demibranch with a single inter-filamentary connection.

Mouth is wide, funnel-liked, followed by a rigid oesophagus. Stomach covered with digestive diverticula; hindgut passes through pericardium (Fig. 2).

The holotype is a hermaphrodite with both testes covering anterior upper quarter of digestive diverticula and the ovaries located posteriorly (Figs 2, 3).

##### Variation.

The shell of the paratype is slightly smaller in absolute dimensions but retains the same ratios of width to length and height to length (Table 1). Like the holotype, the translucent fragile shell is subquadrate; inequivalved with the right valve overlapping the left. In keeping with its smaller size (Allen and Turner 1974), the shell surface has both fewer radial periostracal lamellae (27–29) and secondary lamellae (8–10). The hinge margin is thin and edentate, with a lithodesma that matches the holotype in size and proportions. No silt covering observed.

The mantle of the paratype, as with that of the holotype, has flask-shaped mantle glands. The inhalant siphon is surrounded by 17 papillated tentacles. As in the holotype, there are two left and right pairs of conical tentacles located slightly outside of the row of papillated tentacles. The first pair is located between the second and third papillated tentacles; the second pair is between fifth and sixth (counting from anterior to posterior). The exhalant siphon is surrounded by a total of five conical tentacles; one dorsal and two pairs of laterals. As in the holotype, the foot has a single byssal thread attached.

##### Etymology.

The new species name is derived from the Latin adjective hispida due to the somewhat ‘shaggy’ appearance of the shell.

##### Distribution.

Known only from the type material: Eastern Pacific, southern California, 411–449 m.

##### Habitat.

Type specimens found in silt and fine sand.

## Discussion

The new species was assigned to the genus *Policordia* on the basis of conchological features. The shell surface is smooth, wihtout granules and hinge teeth are absent (Dall et al. 1938; Allen and Turner 1974). Anatomical details of the type species of the genus, *Policordia diomedea*, are not known. Conchologically, the new species differs from the *Policordia diomedea* in that the length is greater than the height and the posterodorsal margin is straighter and longer. *Policordia diomedea* is taller; more rounded with a shorter posterodorsal margin (Dall et al. 1938).

*Policordia hispida* sp. n. most closely resembles *Policordia densicostata* (Locard, 1898); *Policordia pilula* (Pelseneer, 1911) and an undescribed species, *Policordia* sp. (= *Policordia pilula* sensu Ivanova, 1977 not Pelseneer, 1911) (L. Safonova pers. obs. 2016).

*Policordia densicostata*, an Atlantic species whose anatomical features were described by Allen and Turner (1974), has a taller shell with more prominent umbones. *Policordia densicostata* lacks simple conical inhalant tentacles of *Policordia hispida* sp. n. Unlike *Policordia densicostata*, the new species lacks long cylindrical portion of the mantle glands (Table 2).

**Table 2.** Some characters of selected species of *Policordia* including geographical distributions.

	*Policordia hispida* sp. n.^†^	*Policordia densicostata* (Locard, 1898)^‡^	*Policordia pilula* (Pelseneer, 1911)^§^	*Policordia* sp. (un–described)^|^
	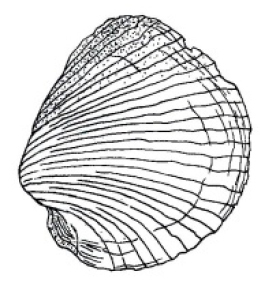	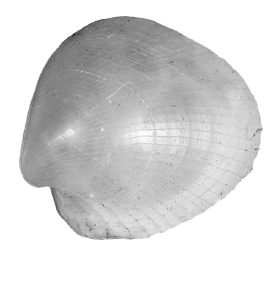	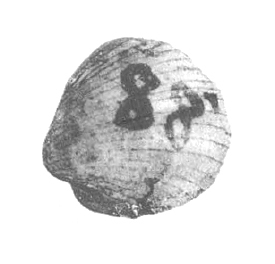	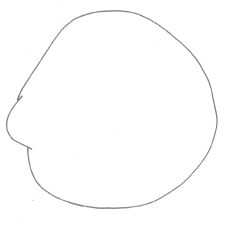
Length (mm)	4.8	18	5.8	7.0
Length:Height	L > H	L < H	L < H	L = H
Inhalant siphon tentacles	18–19, thick, flattened, with 6–8 papillated extensions; 4 outer, smaller, simple conical tentacles	22, all merged at base, each with 3–7 papillated lobes	Unknown	23, thick, flattened, with 7 extensions; 4 outer, smaller, simple conical tentacles
Exhalant siphon tentacles	Conical, 1 dorsal, 4 lateral (left and right pairs)	Conical, blunt with dense covering of papillae, 1 dorsal, 4 lateral (left and right pairs)	Unknown	Conical, 1 dorsal, 4 lateral (left and right pairs)
Byssus	Present	Present	Unknown	Absent
Mantle glands	~ 30; flask shaped without long cylindrical portion	~ 30; flask shaped with long cylindrical portion	Unknown	36–34 flask shaped without long cylindrical portion
Shell sculpture	30–32 radial periostracal lamellae (holotype)	30–37 radiating lines of slightly raised ridges	~ 30 radial hair like lines	~ 30 radial lines
Distribution	Eastern Pacific, Southern Californian Bight; 411–449 m	Atlantic; 1007–2503 m^¶^	West Pacific; 1301 m	Northeastern Pacific, Gulf of Alaska; 1230–2980 m

† Cited herein

‡ Locard 1898; Allen and Turner 1974; Image: Oliver et al. 2010

§ Pelseneer 1911; Prashad 1932; Soot-Ryen 1966; Image: Prashad 1932, fig.24

| *Policordia pilula* of Ivanova 1977 not Pelseneer 1911; Image: Ivanova 1977, fig. 18a

¶ Allen and Morgan 1981; Salas 1996

*Policordia pilula* has a more vertically extended shell with a more rounded posterodorsal margin (Prashad 1932). The general anatomical characters of this species were described by Pelseneer (1911), but he did not provide any details about the siphon and siphonal tentacles (Table 2).

Specimens identified as *Policordia pilula* by Ivanova (1977) from the Gulf of Alaska differ from the original description by Pelseneer (1911) and the subsequent description by Prashad (1932). It is an undescribed species (L. Safonova, pers. obs. 2016). The shell shape of *Policordia hispida* sp. n. is very similar to *Policordia pilula* sensu Ivanova (1977), but differs in the number of radial ribs and mantle glands, the smaller number of tentacles of the inhalant siphon and the presence of a byssus (Table 2).

## Supplementary Material

XML Treatment for Policordia

XML Treatment for Policordia hispida
